# A guidance to intelligent metamaterials and metamaterials intelligence

**DOI:** 10.1038/s41467-025-56122-3

**Published:** 2025-01-29

**Authors:** Chao Qian, Ido Kaminer, Hongsheng Chen

**Affiliations:** 1 ZJU-UIUC Institute, Interdisciplinary Center for Quantum Information, State Key Laboratory of Extreme Photonics and Instrumentation, Zhejiang University, Hangzhou, China; 2 ZJU-Hangzhou Global Science and Technology Innovation Center, Key Lab. of Advanced Micro/Nano Electronic Devices & Smart Systems of Zhejiang, Zhejiang University, Hangzhou, China; 3 Department of Electrical and Computer Engineering, Technion-Israel Institute of Technology, Haifa, Israel

**Keywords:** Materials for optics, Optical materials and structures

## Abstract

The bidirectional interactions between metamaterials and artificial intelligence have recently attracted immense interest to motivate scientists to revisit respective communities, giving rise to the proliferation of intelligent metamaterials and metamaterials intelligence. Owning to the strong nonlinear fitting and generalization ability, artificial intelligence is poised to serve as a materials-savvy surrogate electromagnetic simulator and a high-speed computing nucleus that drives numerous self-driving metamaterial applications, such as invisibility cloak, imaging, detection, and wireless communication. In turn, metamaterials create a versatile electromagnetic manipulator for wave-based analogue computing to be complementary with conventional electronic computing. In this Review, we stand from a unified perspective to review the recent advancements in these two nascent fields. For intelligent metamaterials, we discuss how artificial intelligence, exemplified by deep learning, streamline the photonic design, foster independent working manner, and unearth latent physics. For metamaterials intelligence, we particularly unfold three canonical categories, i.e., wave-based neural network, mathematical operation, and logic operation, all of which directly execute computation, detection, and inference task in physical space. Finally, future challenges and perspectives are pinpointed, including data curation, knowledge migration, and imminent practice-oriented issues, with a great vision of ushering in the free management of entire electromagnetic space.

## Abstract (web summary)

The bidirectional interactions between metamaterials and artificial intelligence have recently attracted much attention. Here, the authors stand from a unified perspective to discuss intelligent metamaterials (AI for metamaterials) and metamaterials intelligence (metamaterials for AI).

## Introduction

The hierarchy of understanding and especially harnessing electromagnetic (EM) waves epitomizes the course of human civilization^1^. As early as two millennia ago, there are archaeological records about focusing sunlight to light a fire using a concave mirror and a lens made of ice^2^. To modern times, relentless explorations have progressively unveiled its mystery, in particular, the intense debate on the relation between electricity and magnetism in the early nineteenth century. This controversy, persisting longer than half a century, is culminated with a unified EM theory formulated by Maxwell, setting a foundation for electromagnetism and optics^3^. The Maxwell’s equations reveal that the capability of manipulating EM waves inherently boils down to the precious control of constitutive parameters, i.e., permittivity ($\overline{\overline{\varepsilon }}$) and permeability ($\overline{\overline{\mu }}$) in tensor form. However, natural materials, in large part, homogeneous and isotropic, is difficult to meet various requirements on constitutive parameters. Therefore, how to surmount this limitation and enrich the existing material library becomes imperative, simultaneously with the pursuit of miniaturization and integration.

Metamaterials, a family of artificially engineered materials composed of subwavelength unit cells (meta-atoms), was born at the end of the twentieth century to shape the landscape of natural materials^4–6^. By mimicking the compositions of natural materials, a dizzying spectrum of EM response can be customized by rationally designing the geometrical structure and spatiotemporal layout. Such liberation greatly motivates scientists to defy standard physical laws and unlock exotic phenomena, such as negative refraction^7^, super-resolution imaging^8^, and invisibility cloak^9,10^. First introduced at microwave, yet, the widespread utilization of metamaterials was hampered by the obstacles related with the fabrication, bulky volume, and high insertion loss. To address this, metasurfaces^11,12^, as the two-dimensional (2D) version of metamaterials, have been suggested as a promising candidate for their unprecedented ability of engineering polarization, amplitude, and phase of EM waves. The features of negligible thickness, better integration, and lower loss make metasurfaces rapidly gain wide popularity and the operation regime has been extended to acoustics, water wave, heat flow, and other physical systems^13–15^. The building blocks can be composed of, such as, subwavelength-scale metallic, dielectric, and semiconducting resonator. Recent progresses have been directed towards tunable modality to allow dynamic manipulation of EM waves^16–18^.

The past two decades have witnessed relentless advancements in metamaterials and metasurfaces, but they still suffer from multifaceted challenges that impede their further off-the-shelf applications. Often the main application shall be oriented to real-life environment and real-time customer demand. This remains out of reach of state-of-the-art metamaterials, because either they are set in stone after fabrication or work in an idealistic environment. Although reconfigurable and programmable metasurfaces have been intensively studied, they require to be guided by external assistance^19^. Setting aside this, other common challenges associated with structural design, narrow bandwidth, sophisticated optical fabrication, and more are waiting to be addressed^20–25^.

**Fig. 1:**
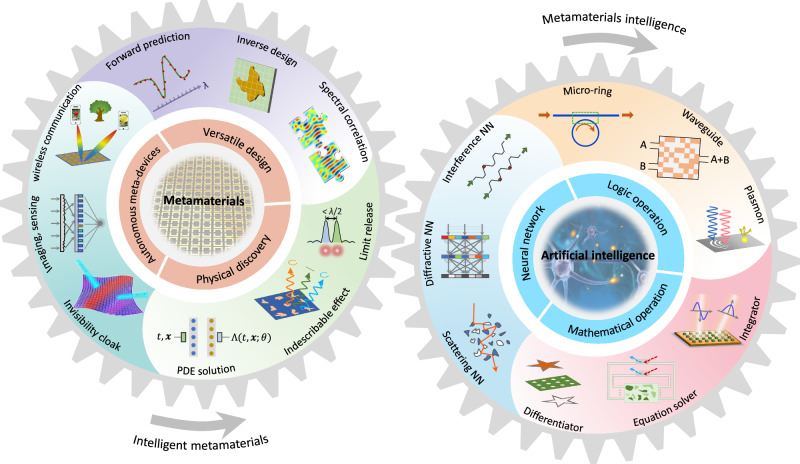
Bidirectional interactions between metamaterials and AI. Metamaterials and AI are like two gears that are meshed together. The prosperity of AI revolutionizes conventional computational methods and working manner of metamaterials, which boils down to intelligent metamaterials. To be specific, intelligent metamaterials are embodied in versatile design, automatic meta-devices, and physical discovery. In turn, metamaterials provide a versatile wave-based platform to execute neural network, mathematical operation, and logic operation at physical space. We conclude this process as metamaterials intelligence because inanimate metamaterials are endowed with the ability of independently computing and analysing. PDE, partial differential equation. NN, neural network.

In parallel, artificial intelligence (AI) has made mind-blowing advances. As the main branch of AI, deep learning allows a computational model to learn representations of data with multiple levels of abstraction and thus carry out tasks without explicit programmed and procedural instructions^26^. The unique advantages of deep learning lie in its data-driven signature to help the model discover useful information from a moderate amount of data with strong nonlinear fitting and generalization ability. Its awakened wave has swept from the mainstream domain of image recognition and language translation^27,28^ to cutting-edge neuroscience and quantum mechanics^29,30^. In the context of metamaterials, deep learning is poised to revolutionize the computational manner and laboratory infrastructural landscapes, manifested in three main aspects, as shown in Fig. 1. First, accelerating metamaterial design^31–39^. Much of the versatility of metamaterials is attributed to the high degrees of freedom in geometrical structure and material diversity. It is thus important to effectively design these variables. This procedure is conventionally carried out via a large number of case-by-case EM numerical simulations, in tandem with bottom-up guidance strategy. Deep learning has spawned new ways of gamifying the design procedure, as it can alleviate the time-consuming, low-efficiency, and experience-prone shortcomings in conventional methods. Second, promoting intelligent meta-devices^40–43^. About it, we have briefly explained the motivation and significance above. Yet, reaching this goal is so difficult because it necessitates interdisciplinary researches in science and engineering to construct a complete set of perception-decision-execution system. Third, capturing latent physical laws^44,45^. Wave-metamaterials interactions are notoriously complicated. Using purely physical methods maybe not enough to elucidate it, such as inscrutable non-linear effect and coupling effect. It would be helpful to unearth these fuzzy physical laws and then push the limits of metasurfaces by invoking deep learning. We summarize these forward interaction manners enabled by deep learning as intelligent metamaterials (AI for metamaterials).

The interaction between metamaterials and AI is not one way but bidirectional. The backward interaction attracts much attention against the background that the existing electronic computers have become increasingly difficult to meet the requirement of processing data with high speed and efficient energy^46^. This dilemma of electronic computers stems from the interconnect delay and large heat generation, inextricably tied to Moore’s law^47^. In this context, metamaterials provide a radically new wave-based computing platform for AI and other information processing. Due to its unique features of signal propagation at the speed of light, low power consumption, and the capability of parallel processing, wave-based computing holds huge potential in a host of practical scenarios, particularly those involving high-throughput and on-the-fly data processing, such as augmented reality and autonomous driving^48,49^. In essence, wave-based computing harnesses the natures of wave dynamics to create the computing isomorphism of different computing tasks. Despite a variety of computing forms, in physics, they fall into three general categories based on EM wave diffraction, scattering, and interference, by virtual of the wave properties of intensity, polarization, frequency, and more. Many architectural concepts and implementations are already under investigation, such as differentiation, convolution, Fourier transform, and random projection^50–54^. From the view of execution tasks, current researches can be classified into three main types, neural network, mathematical operation, and logic operation, as outlined in Fig. 1. We conclude this backward interaction as metamaterials intelligence (metamaterials for AI).

In this Review, we provide an overview of the bidirectional interaction between metamaterials and AI. For intelligent metamaterials, we discuss how to expedite the forward prediction, inverse design, and spectral correlation, develop automatic meta-devices, and capture hidden knowledge that cannot be readily accessed. In particular, we underscore new data augmentation techniques and physical-informed neural networks to alleviate data-intensive requirement and to cope with intractable many-to-many mapping in metamaterials. For metamaterials intelligence, we outline the physical principle and recent achievements of wave-based analogue computing in term of neural network, mathematical operation, and logic operation. In the last part of the Review, we provide an outlook for possible future directions and challenges that encourage cross-pollination between different disciplines to usher in a more intelligent metamaterial era.

## Intelligent metamaterials (AI for metamaterials)

### Three design categories: forward prediction, inverse design, and spectral correlation

#### Forward prediction and inverse design

**Fig. 2:**
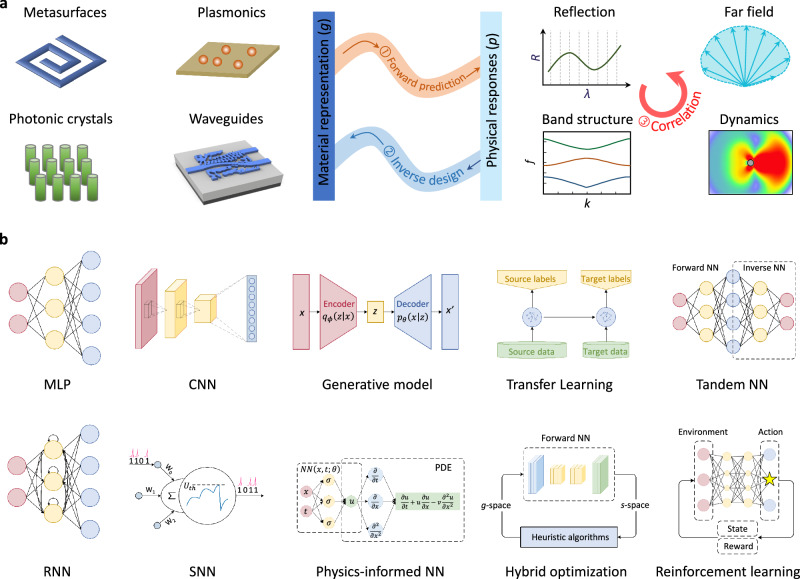
Deep learning based three design categories and orthodox network structures. **a** The first design, forward design, aims to build up a rapid connection from materials representation (*g*- space) to physical responses (*s*-space). The second, inverse design, is opposite to, but much difficult than forward design. The third is to correlate physical responses themselves, such as from low- frequency to high-frequency, from near-field to far-field. **b** Diverse deep learning algorithms serve as surrogate physical models/numerical simulators to accelerate and streamline the design procedure. Apart from conventional neural networks extended from computer science, researches also explore unique networks with ‘local specialty’. MLP, multilayer perceptron. CNN, convolutional neural network. RNN, recurrent neural network. SNN, spiking neural network. “Waveguides” in panel **a** adapted with permission from ref. ^70^, Wiley.

Most problems encountered in metamaterials studies are deciphered through the framework of one of two categories (Fig. 2a). The first is how to derive the EM response (e.g., dispersion feature, near-field distribution, photonic bandgap, radiation pattern, and reflection/transmission) of a given metamaterial, namely, forward prediction. This problem is not difficult to answer as there are a number of analytical methods and numerical modular tools. The second is how to craft suitable metasurfaces for a user-desired EM response, namely, inverse design. The objectives of inverse design are not limited to the often-stated geometrical structure, but covering material diversity, spatial topology, phase profile and, more generally, all physical variables that affect the EM response. Inverse design is opposite to forward prediction, yet, they are not simply reciprocal to each other due to the non-uniqueness issue. And inverse design is much challenging because of the non-convex solution space with many local optima.

For forward prediction, there are two main routes, i.e., analytical method and numerical simulation. The advantage of analytical method lies in the fast computation, but its application scenarios are typically limited to simple, regular, and periodic geometries. For example, the reflection/ transmission coefficient of planar waveguide^55^, multi-layered cylinder/sphere^56,57^, and gradient refractive indices^58^ can be exactly calculated via Mie scattering theory and transformation optics^9^. In addition, approximate model offers a shortcut to attain the simplified characteristics of metamaterials, such as equivalent circuit model that describes metasurfaces using basic lumped elements^59^. In contrast, numerical simulation is much more universal by discretizing the whole solution space into a plethora of subwavelength grids. By setting up sufficient meshes and suitable initial conditions, we can accurately calculate the EM response of a given metasurface. Based on the discretization form of Maxwell’s equations, mature techniques have been developed, such as finite-difference time-domain (FDTD) method^60,61^, finite-element modelling (FEM)^62^, and the method of moments^63^. In each situation, numerical simulation necessities iterative and lengthy calculations of Maxwell’s equations until convergence, especially for three-dimensional (3D) and large-scale scatterer.

For inverse design, its solution cannot be directly evaluated, instead requiring the exploration of a suitable solution from a formidably large design space. For metamaterials with a few design parameters, people can easily search for a top-performing structure via parameter sweeping and fine-tuning, in tandem with empirical guidance. However, this “quasi-brute-force” search can hardly be generalized into a feasible strategy and the resultant design is innately flawed by the trial-and- error manner. In place of this painstaking search, optimization algorithms are widely adopted, including gradient-based approaches (e.g., topology optimization^64–71^ and adjoint method^72–74^) and heuristic algorithms (e.g., binary search^75^, genetic algorithms^76^ and simulated annealing^77–80^). These algorithms need to monitor the output in real time, and then send it back to optimization algorithms to form a closed-loop network. For each new customer-specific target, the network should repeatedly update to search for an optimal solution in a prescribed rule. This process calls for a gargantuan number of EM numerical simulations and inevitably encounters many failed attempts, causing a great waste of computing resources. Probably, the ultimate optimization results and serendipity are satisfactory, but they are limited by the convergence speed, random search, and easily fall into a local optimum.

Breakthroughs in deep learning offers a fresh perspective for forward prediction and inverse design of metasurfaces due to the strong ability of modelling highly nonlinear data relationship^26^. Deep learning has now caused a massive disruption into industry and business; however, its startling success has not appeared overnight. The embryo of deep learning goes as far back as the 1940s, when the multilayer perceptron (MLP) is formulated to imitate the thought process of human brain. Subsequently, deep learning went through a very long and arduous journey, overs the eight-decade period with many significant breakthroughs. A number of reviews have summarized the evolution cycle^27^. In 2006, Geoffrey Hinton, winner of the 2024 Nobel Prize in Physics, coined the term deep learning and invented fast-learning algorithm to train deep neural networks efficiently using greedy layer-wise pretraining strategy^81^. Since then, deep learning experienced an exponential development^82^, as researchers were able to train deeper neural networks than had been possible before, with the performance even outstripping humans, such as the famous game between the computer programme AlphaGo and human champion^83^. The basic architecture consists of multiple layers of neurons connected in series. Information propagates from layer to layer via linear operation, such as matrix multiplication, followed by nonlinear activation function. With the data inputted to the network, data features with higher levels of abstraction are captured from lower-level features, and complex network input-output relations can be fitted. Before putting deep learning into utilization, the synaptic strengths between each layer need to be adequately trained to minimize the loss function via a back-propagation algorithm such as a gradient descent or adaptive optimizer.

The application of deep learning in metasurfaces aims to build up a bidirectional mapping between two physical spaces. The first space (*g*-space) is the working condition (e.g., frequency, polarization, and incident angle), and the physical variables that describe the metamaterials. The second space (*s*-space) is the physical EM responses. For forward prediction, deep learning serves as a surrogate model of EM solver to build up a forward mapping from the *g*-space to the *s*-space. This is easy to reach because the mapping is a single-valued function (one-to-one). For instance, a multi-layered sphere with deterministic geometric and material configuration only results in a single transmission spectrum. For inverse design, the idea of directly flipping the input and output side with the neural network unchanged sounds naive due to the one-to-many mapping^84–86^, i.e., non-uniqueness phenomenon. It thus has an incentive to explore more advanced network structures. Deep learning features several core strengths, spawning it complementary to or superior to conventional methods. First, a well-trained neural network can produce flavour-selective results orders of magnitude faster. It merely entails one-single forward computation and navigate vast parameter spaces than data screening or intuition. Second, the strong nonlinear fitting and generalization ability allows deep learning to accurately unlock non-intuitive data relationship and establish physical correlation that cannot be easily accessed by other optimization algorithms. Third, in conventional trial-and-error metamaterial design, tremendous data are disposable and discarded, causing a considerable waste of computational resources and human labour. Deep learning can recycle the seemly-useless data to improve data utilization efficiency^87–90^.

Initial introductions of the neural network into modelling EM devices trace back to the early 1990s in the microwave community^91–97^. Neural networks are first trained to model the electrical behaviour of radio-frequency (RF) components/circuits and then used as a computer-aided design tool for fast determination of the parameters^95^. As an attractive alternative to numerical methods and empirical models, neural network techniques have been adopted in a wide variety of microwave designs, including coplanar waveguide components^91^, microstrip line^92^, filter^96^, and frequency-selective surface (FSS)^97^. At that time, the used neural networks have shallow expression ability, yet, they showcase the preliminary potential in solving EM problems. After about two decades, the epoch-making achievements in deep learning greatly impact electromagnetics and optics. The advent of new training, regularization techniques, and new hardware architectures make it possible to formulate deeper neural networks and describe the high input degrees of freedom^98,99^. Far beyond microwave devices, the design scope has been expanded to the field of metamaterials, metasurfaces, photonic crystals, and plasmonics^100–123^. To capture in more detail how deep networks are implemented, we discuss representative examples in Box 1.

Whatever type of metamaterial designs, two key problems must be deciphered, i.e., labelled data collection and algorithm modelling. Data to deep learning is like the fuel to an engine. To enable a powerful driving force, much effort has been inaugurated to assemble voluminous curated data sets by crowd-sourced annotation. However, it was soon realized that such enumerative method is prohibitively time-consuming and resource-intensive, especially with the increase of design dimension and metamaterial scale. To mitigate this dilemma, data clustering, feature extraction, de-noising, and other related techniques have been extensively leveraged to augment the data utilization efficiency^124–126^. Distinct from image, text, audio, and video applications, a very fortunate point for metasurfaces-related works is that metasurfaces are moderately governed by certain physical laws and semi-analytical solutions. Hence, injecting physical insights has been a highly effective strategy in solving data-scare settings, such as physics-informed training methods to reduce reliance on annotated data^127–129^. In Box 2, we summarize main methods to enhance data utilization efficiency in the context of metamaterials. Another point we want to emphasize is that alleviating data-intensive requirements from the pure view of data maybe not enough, it is also inseparable with network structures, which we will discuss later.

Advanced network structure plays a pivotal role to improve design result, reduce data recourse, and adapt to complex scene. Many deep learning algorithms have been generalized from computer science into the community of metamaterials, such as MLP, CNN, RNN, transfer learning, and reinforcement learning (Fig. 2b). They have been substantially studied with respective strengths and different scenes. For example, CNN is an ideal candidate to process high-dimensional data by performing cross-correlation operation between the incoming tensor and the convolutional kernel. Such operation is architecturally translation-invariant, making it suitable for input images with strong spatial correlation, such as metasurfaces pattern and near-field distribution. RNN is often used to handle time-sequential problems such as transient optical signals and wave propagation in the time domain, because it maintains a memory for the past processed information. Readers interested in these orthodox algorithms and canonical applications can refer to a number of recent reviews^31–36^. To optimize the neural network, a loss function should be defined to describe the difference between the outputted and desired response, such as mean squared error (MSE) and mean absolute error (MAE) for continuous prediction tasks, cross entropy loss and hinge loss for discrete prediction tasks. For illustrative purposes, cosine similarity, Pearson’s correlation coefficient, and structural similarity (SSIM) can be applied to quantify the similarity between the outputted and desired response. To alleviate the problem of falling into a local optimum, a suitable optimization method is crucial. For example, momentum optimization helps to overcome local minima and cross plateaus by accumulating a small fraction of past gradient momentum. Stochastic gradient optimization updates model parameters using a small random subset of the training data. It involves cyclically changing the learning rate between a low and high bound, allowing the model to jump out of shallow local minima. Looking ahead, it would be judicious to forge physical insights into algorithms to make them more unique and exclusive, rather than ‘black box’ treatment and a form of “alchemy” without enough discernments on parsing internal physical mechanism. We would like to draw attention to a few new advancements with ‘local specialty’ forthwith.

#### Deep generative models

**Fig. 3:**
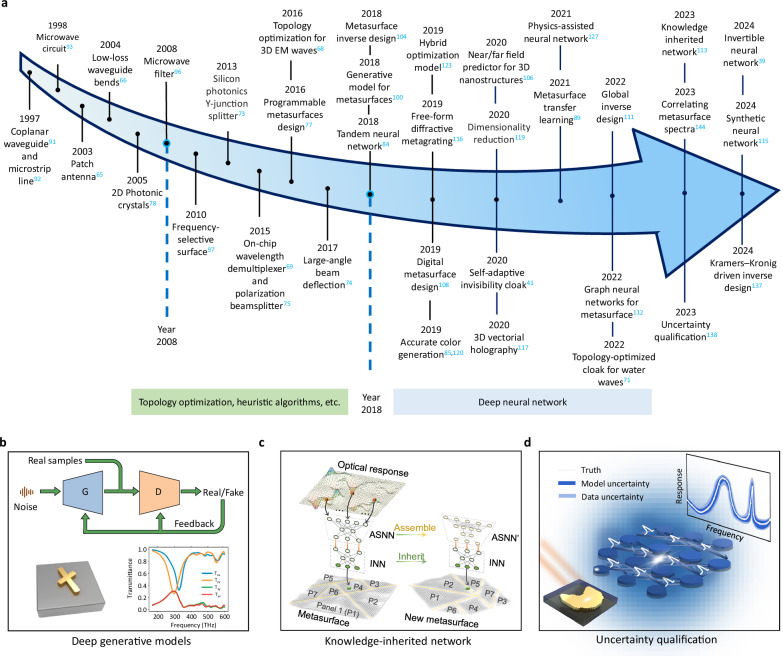
Timeline of advances in metasurface design and related technologies. **a** Initial studies on the design of EM and photonic devices can be traced back to 1990s, starting from the optimization of RF components. In that period, the widely-used algorithms include topology optimization, heuristic algorithms, and simple neural networks. After 2018, deep neural networks have widely penetrated into the design of metasurfaces, photonic crystals, and plasmonics. **b** Generative adversarial network generates candidate metasurface patterns that match the on-demand spectra with high fidelity. **c** Knowledge -inherited network is oriented for multi-object and shape-unbound metasurfaces. Each inherited neural network (INN) carries knowledge from the “parent” metasurfaces and then is assembled to construct the “offspring” metasurfaces with assembled neural network (ASNN). **d** Uncertainty qualification of metasurface design by amendatory Bayesian neural network. Panel **b** adapted with permission from ref. ^100^, ACS. Panel **c** adapted from ref. ^113^, CC BY 4.0.

Generative models have been one of the most hotly researched fields in AI and much of the probabilistic foundations and learning algorithms have been established in the past decade, such as autoregressive models^130^, variational autoencoders (VAE)^131^, generative adversarial network (GAN)^100,101^, and flow-based models^132^. One representative example is the generation of realistic-looking images, voices, or movies; so-called deep fakes. Unlike discriminative network, generative network aims at obtaining the intractable probability distribution between the input and output, rather than single-valued mapping. When trained successfully, we can use deep generative models to estimate the likelihood of a given sample and to create new samples that are similar to samples from the unknown distribution. Such property is well fitted in the field of metasurfaces, because it effectively handles one-to-many mapping in inverse design and provides user with diverse candidates. Among these deep generative models, VAE and GAN stand out, which are widely used in chiral metasurfaces, diffractive grating, and mutual coupling effect prediction. VAE basically contains an encoder that learns a low-dimensional latent representation of the training data called latent variable and a decoder that inversely utilizes latent space to regenerate the images similar to the dataset. GAN is an unsupervised learning that can be trained to achieve an equilibrium by the competition between generator and discriminator networks (Fig. 3b). In addition, diffusion models, as another deep generative model inspired by non-equilibrium thermodynamics, have recently attracted much attention^133^. They define a Markov chain of diffusion steps to slowly add random noise to data and then learn to reverse the diffusion process to construct desired data samples from the noise^134^. We anticipate that the diffusion model will likely find more utilizations in metamaterials by harnessing the advantages of high-quality synthesis, strong diversity, and mode coverage.

#### Knowledge-inherited paradigm

Recently, Jia et al. proposed a knowledge-inherited paradigm or termed as synthetic neural network that allows knowledge inheritance from “parent” metasurfaces to “offspring” metasurfaces^113^, as displayed in Fig. 3c. “Parent” metasurfaces are collected in advance, each of which carries a “parent” neural network. For a given “offspring” metasurface, “parent” metasurfaces can be freely assembled to construct it in physical space (as simple as building a container-type house), corresponding to the dynamic synthesis of “parent” neural networks in network space. The biggest advantage of the knowledge-inherited paradigm lies in the ability of saving a gigantic amount of training data and working for multi-object, shape-unbound metasurfaces design. This is completely different from transfer learning^87–90^. The basic principle of transfer learning is to transfer the weights inside a pre-trained neural network in the source task to assist the training of target task. It heavily relies on the brute-force attack of features and falls short in reasonable explanation. On the other hand, the improvement of transfer learning is incredibly limited, stochastic, and even worse than that without transfer learning. For example, given the same amount of given data in the same task, the loss by transfer learning is non-convergent (28.7% accuracy), while the accuracy reaches 86.7% using the knowledge-inherited learning^113^. Recently, synthetic neural network is further generalized for subwavelength meta-atom and reconfigurable metasurfaces^114,115^.

#### Explainable and causal neural network

Explainability and causality become desired properties in AI assisted metamaterials design. Local interpretable model-agnostic explanations (LIME) and Shapley additive explanations (SHAP) are two widely used techniques to provide deep insights in the decision process. LIME explains the black box’s prediction by learning an interpretable model locally around the prediction^135^. SHAP, a game-theory-inspired method, offers the interpretability by computing importance values for prediction features^136^. To enhance the causality, a new physical adversary channel was created by embedding a universal Kramers–Kronig (KK) relation into deep learning^137^. The superiority of KK-driven casual neural network in forward prediction and inverse metasurface design was demonstrated via modifying loss function and probability distribution in latent space. The exceptional outcome suggests that the similarity between output and given spectra reaches 99.8% and maintains an extremely high fidelity even in mutant band.

#### Uncertainty quantification

“Uncertainty is the only certainty there is,” wrote mathematician John Allen Paulos. Applied in deep learning-related studies, uncertainty is like a parasite coexisting in the incredible outcome^138–140^. This is especially announced in the community of metasurfaces due to the ubiquitous uncertainties from realistic fabrication error and network modelling. Having a prophetic ability to evaluate the uncertainty is important to help users critically face the output results. Although Bayesian approximation and ensemble techniques have been advocated for reliability analysis by replacing the deterministic weights in the network with the probability distributions^141^, these methods are mostly applicable for situations where the input and output data are homologous. Recently, concerning the complicated metasurface dispersion, conventional Bayesian network was revised by embedding physical-inspired elements to evade loss explosion and accommodate non-homologous situations^138^, as shown in Fig. 3d. In the results, the amendatory Bayesian network can simultaneously yield the predicted results and specific uncertainty, providing experimental reliability of different metasurface manufacturers. The outcomes are critical for highly-sensitive optical materials that have never been revealed before.

Relentless efforts have been made to solve a vexing problem in inverse design, i.e., non-uniqueness, meaning that wildly divergent designs produce nearly identical EM properties. In this case, deep learning algorithms will become conflicted on how to adjust learnable weights, making the training process difficult to converge. Here, we systematically summarize a diverse set of strategies for this problem. First, tandem neural network alleviates the non-uniqueness issue by attaching a forward modelling network to the terminal of an inverse design network^84–86^. As such, the inverse design network can be steered to a correct convergence direction with the constriction of the forward modelling network. As is often the case, the weights in the inverse design network still faces conflicting gradients that hinder effective converging. Second, deep generative models offer a probabilistic viewpoint to create a variety of candidate design geometries^100,105^. For example, VAE compresses input and output data into a latent space, which is then sampled by a generative model^105^. Third, hybrid optimization package comprises a discriminative network as the surrogate of EM numerical solvers and an iterative optimization algorithm, such as Newton’s methods and heuristic algorithms^88,122,123^. For each given EM response, the closed-form framework can rapidly generate many candidates by modifying initial condition, adding disturbance and practical limitation. Compared with direct optimization using conventional full-wave numerical solvers, the hybrid package also outputs the results orders of magnitude faster.

#### Spectral correlation

**Fig. 4:**
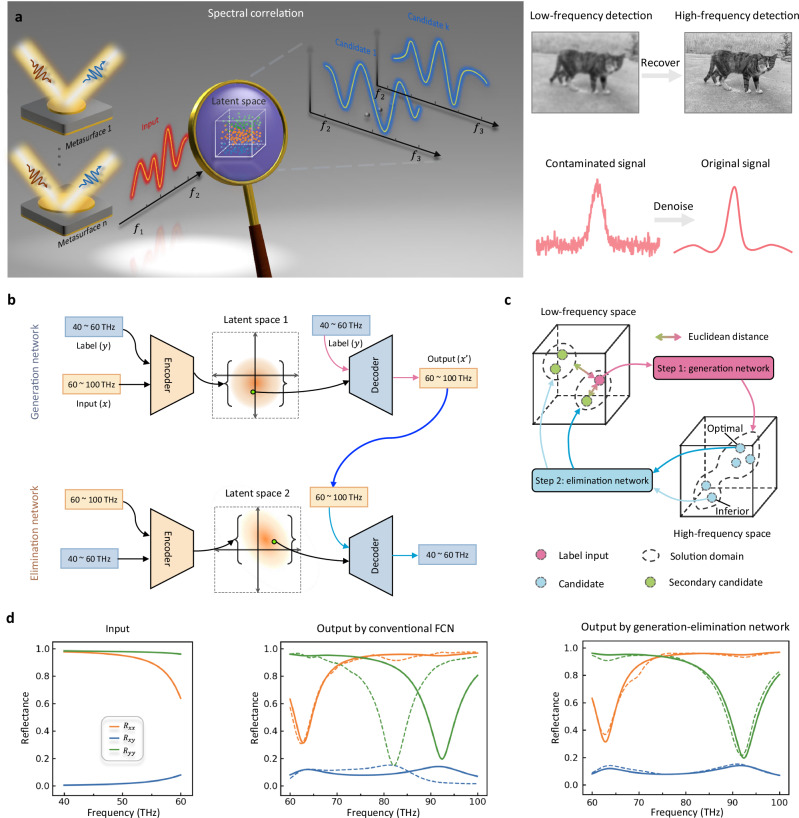
Metasurface spectral correlation. **a** Inferring optical response from low-frequency to high-frequency band is useful but challenging due to the bidirectional non-uniqueness predicament. Such correlation is applicable for recovering high-resolution image and reducing high-frequency simulation time with dense meshes. **b** To solve this, generation-elimination network consists of two cascaded networks is proposed, each of which is composed of an encoder, the latent space, and a decoder. **c** For a given input (low frequency), the generation network can generate various candidates (high frequency), and the elimination network will reversely map each candidate into the original space. The optimal candidate is picked out by calculating the Euclidean distance between the input and secondary candidates. **d** Design example of terahertz metasurfaces. FCN, fully connected network. Panels **a**–**d** adapted from ref. ^144^, CC BY 4.0.

Apart from the widely-studied forward prediction and inverse design, there is another class of metasurface design that has been largely ignored, i.e., inferring optical response from other correlated optical response (Fig. 4). This is instrumental in a wide range of applications, for example, to extract the desired images, spectra, and material features from other easily accessible information with the advantages of low cost and easy measurement^142,143^. Achieving spectral correlation faces a formidable challenge because it involves complex many-to-many mapping, overriding one-to-one and one-to-many mapping. Many-to-many mapping means that there are multiple correct answers for one given input and vice versa. To this end, Chen and co-workers recently proposed a generation-elimination framework to correlate metasurface spectra^144^. The generation network is capable of producing diverse candidates by sampling over its latent space based on a VAE structure. Then, the elimination network eliminates all inferior candidates through the merging of two latent spaces; the operation process is similar to a hierarchical bifurcating tree. Taking for instance the terahertz, the reflection spectra are successfully translated from low to high frequencies without consulting in metasurfaces structural information. The method is meaningful to avoid expensive detection and reduce the simulation time at high frequency, which is also applicable for inverse design with semi-known input^145^. Moreover, it provides interpretable perspectives for deep learning to parse complex physics and facilitate the applications involving cross-wavelength information correlation^146–148^.

Box 1 Examples of deep learning enabled metasurfaces design **MLP**. Although MLP has a rather simple architecture, it has been theoretically proved to be capable of approximating any continuous function arbitrarily well. In the context of metasurfaces, MLP is widely used as a preliminary attempt to uncover light-matter interaction with a limited number of degrees of freedom. The demonstrations to date are many, including topological photonics, chiral metasurfaces, and integrated silicon photonics^103,104^. In spite of a variety of physical scenes, the three-step flowchart is universal, i.e., structural parameterization, data collection, and network training. For example, a four-layer fully connected neural network was used to approximate light scattering by multilayer nanoparticles consisting of alternating lossless silica and titania shells^103^. For a given nanoparticle with different shell thicknesses, the trained network can match the scattering cross section with high accuracy. Moreover, the work offers clear evidence to illustrate that the network indeed learns the physics of the system, rather than simple data interpolation.

**CNN**. An encoder-decoder structure typically comprises convolutional layers, pooling layers, batch normalization layers, and other many basic layer structures. Owing to its hierarchical structure, such network structure has the merits of rapid inference, strong generalization, translation, and scale invariance. A CNN model, comprising two bidirectional neural networks assembled by a partial stacking strategy, was proposed to design individual chiral meta-atom that can solve three basic tasks simultaneously^110^. Furthermore, CNN is used to map the input near-field distribution to global metasurface constellation^111^. The input has three channels for the RGB near-field amplitude image and one channel for the frequency. The output is the distribution of metasurfaces. To facilitate the network training, a cross-entropy loss function is defined as the loss function to backpropagate the gradient of the loss value. The authors benchmarked the global inverse design for on-site illusion customization, achieving the accuracy of 87%.

**Transfer learning**. Data-hungry is an inherent nature for deep learning. Yet, data collection typically requires lengthy simulations and occupies excessive computing resources. Thus, decreasing the reliance on the amount of data is a much-sought goal. Transfer learning is a possible candidate to mitigate this dilemma by using the data in source task to assist the network training in target task^87–90^. Recently, a heterogeneous and transferrable network framework^88^ was proposed for diverse metasurface design, which integrates feature augmentation and dimensionality reduction. Besides basic profits brought by transfer learning, one distinct feature is to generalize transfer learning into more complicate scenes with dimensional mismatch between source and target metasurfaces. The authors demonstrated the feasibility via two scenarios, i.e., metasurfaces with different parameterizations and geometries, where the relative error can be decreased by 23%.

**Reinforcement learning**. The main idea of reinforcement learning is to enable an agent to assimilate the parameter space of an environment from its own actions and experiences^120,121^. Current metasurfaces applications include structural colour generation, matrix multiplier and more. Rho’s group used deep Q-learning to optimize the morphology of silicon nano-disk based on the matching level between the reflection spectrum and a given colour standard. It turns out that the model can find the closest representation of RGB from millions of possible states within 9,000 steps, such as much purer red, green and blue colours compared to previously reported results^120^.

**Hybrid optimization model**. By reconciling conventional optimization methods with advanced deep learning algorithms, it is possible to unleash respective advantages and largely shake off the high demand on computational resources. In the hybrid model, deep learning algorithm serves as a high-speed agent of EM numerical simulator. For example, by connecting a trained forward neural network with a heuristic algorithm (e.g., a genetic algorithm), the closed-form system can rapidly generate the candidate metasurfaces after a number of iterations^88,122,123^. In most cases, we can flexibly embed practical limitations and customer-favoured policy into the optimization. Another advantage is that such hybrid model can provide user with more-than-one possible solutions and thus addressing the one-to-many mapping in inverse problem.

**Tandem neural network**. In inverse design, the existing non-uniqueness phenomenon makes most of inverse-design neural network ill-posed. To relieve this problem, Liu et al. proposed a tandem neural network by connecting the input of a forward network to the output of an inverse neural network^84^. The forward network is firstly trained and then fixed to force the inverse network to converge towards the correct direction. If the convergence does occur, the network alleviates the non-uniqueness issue but without the guarantee of completely solving it. Once proposed, this method has quickly extended into a variety of physical scenes. For example, Zhen and colleagues skilfully solved the design of two-layer transmitted metasurface cloak with 16 free variables^86^.

Box 2 How to help neural network adapt to high-dimensional metasurfaces input? For a design object with a low-dimensional design space, brute-force data collection and conventional algorithm modelling are computationally feasible. However, with the increase of dimensionality of the design space, the solution space will become unfathomably vast, making the problem extremely tricky. To relieve this, we may start from three aspects.

First, data augmentation is a widely-used technique of increasing the size of data with low cost. In computer vision, the most common data augmentation techniques include position augmentation (e.g., scaling, cropping, and flipping) and colour augmentation (e.g., brightness, hue, contrast). A portion of these techniques are possible to be extended into the metasurface community. However, we want to stress more special data augmentation by imbuing physical insight, which has been rarely studied. For example, transformation optics offers great versatility to control the trajectories of light^279^. The underlying mechanism stems from the formal invariance of Maxwell’s equations: a coordinate transformation does not change the form of Maxwell’s equations, but only changes the constitutive parameters and field values. Before and after transformation, the optical response can maintain the same, while the EM constitutive parameters can be various. In this way, we may generate numerous variants to speed up data collection. Similar idea can be implemented by introducing equivalent physical models into metasurfaces.

Second, reformatting the input/output data plays a decisive role in whether the neural network can readily “understand” the data. For example, in the context of metasurface, most of works consider the amplitude and phase of the reflection/transmission spectra as input. However, due to the abrupt wiggles existing at resonant frequencies, it is a big barrier for neural network to grasp the intricate relationship. In contrast, we represent them into the real and imaginary parts as output to make the curves much smoother for easy training. refs. ^88,280^. has clearly shown the great improvement by using such input format. Other examples include transforming a binary image/data string from real space into frequency domain with sparse representation by using Fourier transform /discrete cosine transform^88^. Such transform can condense the information to a compact space, which is helpful to reduce complexity and prevent overfitting.

Third, dimensionality reduction means to project a high-dimensional space into a low-dimensional space, and the popular methods include principal component analysis (PCA) and autoencoder (AE). PCA starts from a statistical view to increase data interpretability but at the same time minimize information loss^281^. The key step is to identify a new orthogonal basis in the high-dimensional space, according to the sequential specification of basis vectors with maximal component scores. AE contains an encoder and a decoder. The encoder maps the input data to a low-dimensional space, called as latent vector, while the decoder network maps it back to the high-dimensional space. In a multilayer thin-film structures composed of consecutive layers of silica and titania, AE based method shows two to three orders of magnitude reduction in the required computation, compared with conventional neural network without dimensionality reduction^282^.
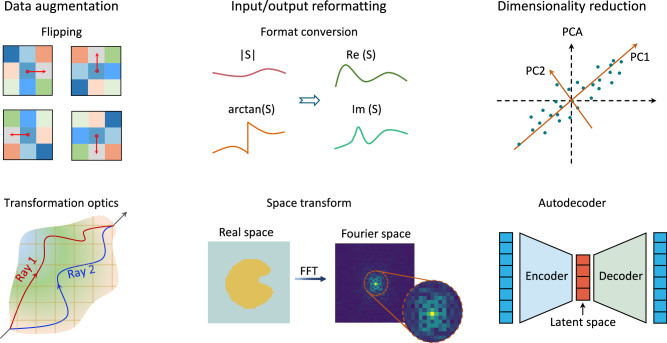

### Intelligent meta-devices

Metamaterials and metasurfaces have stimulated abundant applications in the past two decades, such as invisibility cloaks, holograms, planar optical lenses, polarization converters, absorbers, and vortex-beam generators^41–43,149–153^. Operating frequencies have also been generalized across microwave, terahertz, and optical regimes by designing suitable micro-/nano-structures, covering all one, two, and three dimensions. Despite a wide class of meta-devices, most of them work in a passive fashion with fixed functionality and for a specific incident wave that was assumed a priori known. Against this context, flexibly tuning metamaterials properties is highly demanded for practical applications, giving rise to the proliferation of programmable and reconfigurable metasurfaces. They have been extensively studied and underpinned the performance of the established technologies, but they are encountered with a knotty issue of how to quickly and automatically cater for customer-defined functionalities and on-site requirements^40^.

**Fig. 5:**
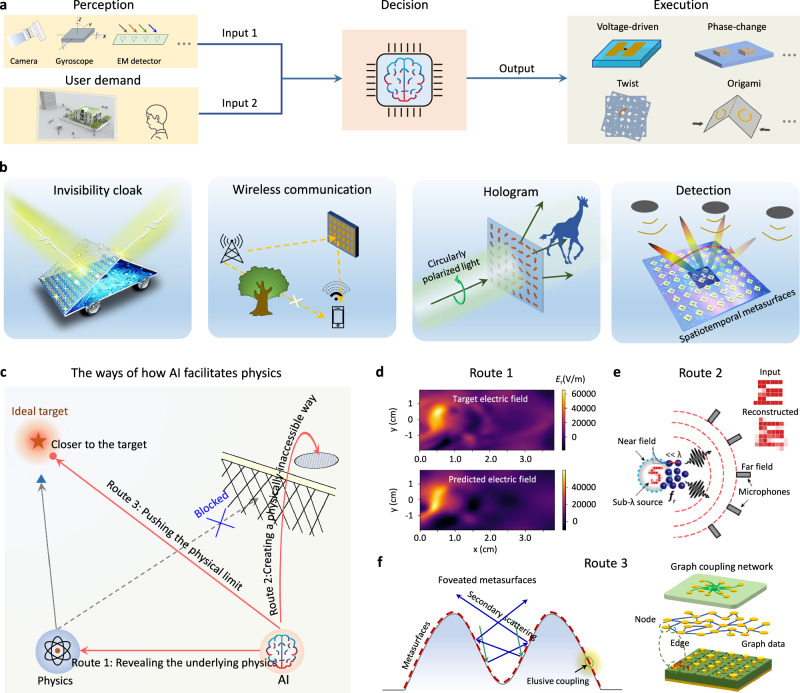
Intelligent self-adaptive meta-devices and renewed physics. **a** A universal architecture of intelligent self-adaptive meta-devices integrated with perception-decision-execution. The dynamic changes of external environment and meta-devices themselves are monitored in real time to feed into AI-driven decision module, together with user demand. Then, reconfigurable metasurfaces execute the command outputted by decision module. **b** Representative meta-devices applications of deep learning. **c** AI facilitate physics in three routes. **d** Uncovering edge plasma dynamics using physics-informed neural networks (PINNs). **e** By placing a locally resonant metamaterial in the near field, the imaging information can be encoded in the far field, enabling the reconstruction and classification of the digits by the neural network. **f** Pushing the limit of metasurface cloak in terms of working bandwidth, free-form shape, and incident angle. The left shows the reflection efficacy improvement with near-field coupling effects taken into account. Panel **d** adapted with permission from ref. ^194^, APS. Panel **e** adapted with permission from ref. ^188^, CC BY 4.0. Panel **f** adapted with permission from ref. ^159,160^, Wiley.

Endowing metamaterials with intelligence is undisputedly of central importance to self-adapt to practical surrounding environment and external stimuli. Nowadays, intelligent meta-devices are a fashionable area of research, making it difficult to separate the hype from the true utility. What are intelligent meta-devices? Although the definition is vague, experts generally agree that the iconic metric is the ability of intelligently sculpting EM waves to perform complex task without human participation. To this goal, intelligent meta-devices shall have three crucial modules, perception, decision, and action, which are systematized in a closed-loop or open-loop network (Fig. 5a). The key difference is feedback. A closed-loop system monitors the ad hoc output in real time and alters it to the desired one via the decision and action modules^152,153^. An open-loop system make action based on the input from perception module. In the context of intelligent meta-devices, perception module typically includes surrounding environment (e.g., urban building and indoor circumstance), external stimuli (e.g., EM wave and multi-physical field), and meta-devices themselves (e.g., gesture and moving speed)^154–156^. For a closed-loop system, the complexity of perception module is largely lowed, because it only senses and feedbacks the detected EM signals.

Decision module is like a brain to decide how to command action module. To put it deeply, decision module builds a connection between the global EM response and meta-devices. Although the intelligent design of individual meta-atom has been well studied thus far, sometimes, it is not accurate enough to characterize the overall EM response of a meta-device by certain geometric principles, such as Fermat’s principle^157^, antenna theory^18^, and effective homogeneous method^158^. This is because the constituent meta-atoms are not independent but coupled with each other. Moreover, for a freeform meta-device, second-, third-, or even more high-order scattered waves may happen, making the problem more elusive^159,160^. In light of these factors, streamlining the global design of a meta-device is highly demanded to enable the decision module, a necessary condition to propel the next generation of intelligent metamaterials and meta-devices^40^. Compared with the design of a single unit cell or small-scale metamaterial, a formidable difficulty lies ahead in the increase in the solution space’s dimensionality. If one purely treats this situation as a black box with orthodox algorithms, the problem will become unsolvable and inextricable. Overcoming this difficulty puts forward a very high requirement on data generation and curation and physical-based network modelling. Integrating fundamental physical laws and domain knowledge into deep learning is a very promising road by ‘teaching’ deep learning about the physical constraints that is unrelated with observational data^107,137^.

Action module, as implied by the name, is a physical platform that executes the commands from decision module. Passive metasurfaces are a non-optimal option because they can only execute a single fixed functionality after fabrication. To enable a dynamical control of EM waves, tunable, reconfigurable, switchable, and active metasurfaces are favoured^161–170^. Different spectral regimes have different tuning means, classified into two major categories. One is based on mechanical actuation. For example, micro-electro-mechanical systems (MEMS) and origami/kirigami methods bestow metasurfaces with the ability of reconfiguring the resonator shape and spatial arrangement^161–163,168^. Stretchable substrates based on polydimethylsiloxane (PDMS) is able to adjust the lattice constant for the applications of varifocal metasurface lens, dynamic hologram, and anomalous refraction^169^. The other is achieved by incorporating active components, including varactor diodes, phase-change materials, semiconductors, and liquid crystals, either as surrounding dielectric environment or as constituent materials^165,166^. Stimulated by pumping light, external magnetic field, and voltage, etc., these active components make response at a fast speed to change the permittivity, topological state, surface impedance, and reflection/transmission spectra of metasurfaces. Recently, temporal dimension has been imbued into metasurfaces to enable time-varying or spatiotemporal metasurfaces. Maxwell’s equations suggests that such introduction breaks time-reversal symmetry and Lorentz reciprocity and evokes many new physical discoveries, including inverse Doppler effect and frequency shift^16,171–174^. These intriguing features provide a versatile action module to manipulate EM waves in both space and frequency domains.

Having the above three modules, a myriad of exotic applications would be anticipated (Fig. 5b). Below, we will talk about the representative advancements in cloaking and wireless communication.

#### Invisibility cloak

Becoming invisible at will has fascinated humanity for millennia, as it is of prime significance for fundamental science and vast applications in spacecraft components and electronic shielding. From a physical point of view, the essence of invisibility cloak is to suppress the scattering strength of a hidden object or disguise the scattering characteristic as other objects. EM absorber is an early cloaking technique^175^. It neither reflects nor transmits incoming waves but instead largely absorbs incoming waves with special materials, such as carbon materials, metal fibres, and ceramic materials. Nevertheless, absorber works for single base station detection and “black” background (e.g., an aircraft in the sky) that hinders the wide applications. In 2006, the advent of transformation optics paves a new avenue for modern invisibility cloak^9^. Based on the formal invariance of Maxwell’s equations, transformation optics render an object invisible by guiding the flow of light around the hidden object without disturbance to the internal object. Soon after, scientists carried out proof-of-concept experimental validation cloaking phenomena, such as a simplified 2D cylindrical cloak made of split-ring resonators in the microwave. Other invisibility methodologies, such as scattering cancellation and scattering reconstruction, have also been proposed for specific scenario in the regimes of EM waves, sound, elastic waves, and heat flows.

However, existing cloaks mostly work in a static environment and predefined EM waves. In reaction to an external stimulus or non-stationary environment, an ideal invisibility cloak should rapidly and automatically adjust its internal active structure to remain invisible at all times. Recently, Qian et al. presented a chameleon-like intelligent invisibility cloak that can rapidly self-adapt to the ever- changing background environment and external stimuli without human intervention^41^. The reflection property of each element inside the metasurface can be independently tuned by feeding different bias voltages across a loaded varactor diode at microwave frequencies. In the proof-of-concept microwave experiment, a set of perception-response-cloaking system was built up to imitate a real-world scene and fully benchmark its millisecond self-adaptability and robustness. After that, intelligent cloaks are followed in the form of EM illusion^111^, and very recently, they are advanced into three-dimensional vehicle and flying cloaked drone^176–178^. These works bring the available cloaking strategies closer to practical scenarios involving changing backgrounds, moving objects, and multi-static detection, moving cloaking research into its next stage—intelligent cloaks.

#### Wireless communication

Recent trend in wireless communication has advanced towards the realization of ultra-high data rates, low energy consumptions, global coverage, reliable connectivity, and low latency. The fifth generation (5 G) is the latest attempt that brings mobile communication up to speed to satisfy the requirement for the next 10 years. The 5 G technique primarily applies massive multiple-input multiple-output (MIMO) and orthogonal frequency-division multiplexing (OFDM) that divides frequency dimension into multiple orthogonal channels for parallel transmission^179^. To this goal, conventional approaches mainly include deploying more active nodes, such as base stations, access points, relays, and distributed antennas/remote radio heads, to shorten the communication distance for achieving enhanced network coverage and capacity. However, this may incur a high energy consumption and deployment/backhaul/maintenance cost, as well as more severe and complicated network interference issues. Wireless communication based on metasurfaces, also known as intelligent reflection surface (RIS), holds considerable potential to make advancements in 5 G technique and the construction of smart cities^180^. Compared with conventional phased array or other active wireless relays, metasurfaces greatly relieve the heavy reliance on high-complexity, energy-consuming radio-frequency hardware at base stations^181–186^. In contrast, metasurfaces manipulate the received signal in a passive manner without complex active transmit modules (e.g., power amplifier). It thus allows intensive deployment of communication equipment convenient in a green manner. There are an increasing number of works about using metasurfaces for wireless communications. For example, Fan et al. introduced the concept of homoeostatic neuro-metasurfaces to automatically and monolithically manage wireless channel in the dynamic propagation^185^. A mechanical-actuating neuro- metasurface is proposed, and for each neuro-element, the reflection phase is separately tuned by mechanical rotation. Mechanical neuro-metasurfaces execute geometric actuation only in one step without continuous energy supply (non-volatile); the power consumption plummets. In addition, the heat dissipation issue could be relieved to some extent, and the anti-jamming capability could be lifted in volatile environments^187^. Even more speculatively, the study of neuro-metasurfaces is promising for many other applications, including target localization and electronic surveillance.

### Physical discovery

Other than the above two main areas, the connation of intelligent metamaterials has penetrated into scientific and industrial domains. The more promising results reported, the more questions will be raised. How to remarkably reduce the volume of the training data? Can artificial intelligence challenge the existing physical limit? Can artificial intelligence really capture latent physics that have even not realized yet? Is there any nuance in training process or like medieval alchemists? We are still on the way of resolving these questions to set the foundations. Many stirring applications and yet-to-be-conceived concepts are waiting to be unearthed (Fig. 5c).

A category of researches introduces deep learning to push the performance of meta-devices that are fundamentally governed by physical laws, such as diffraction limit. We emphasize that although deep learning holds the potential to push the performance beyond the existing methods, it does not mean that deep learning can break these physical laws as they are strictly limited in theory. For example, to display the sub-diffraction imaging details, conventional methods require to utilize the evanescent wave components. Unfortunately, the evanescent wave cannot propagate far, inevitably limiting the imaging resolution. To solve this, Fleury group combined resonant meta-lens and neural network to recover the details as small as λ/30^188^. By placing a locally resonant meta-lens in the near- field region, the information can be encoded in the far field, further fed into neural network to inversely reconstruct the image (Fig. 5e). In optical information storage, Wiecha and collaborators trained deep neural network for the optical retrieval of digital information encoded in the geometry to achieve quasi-error-free readout of sequences of up to 9 bits^189^. Other examples are many, including overcoming the limitation of camera-based systems by a radio frequency (RF) based Lip-reading framework^190^, and introducing end-to-end framework to fully explore the prescribed design space and push the multifunctional design capacity^159,160,191^, as shown in Fig. 5f.

Another category of researches is to solve the governing equations that describe the behaviour of complex physical systems^192–195^. Deep learning serves as an intermediate step to solve partial differential equations and wave equations in a generalized framework. For example, PINNs discover governing partial differential equations from scarce and noisy data for nonlinear spatiotemporal systems and acoustic wave equation for spatially-varying velocity models^195^. In these examples, we do find the great potential of deep learning in fitting/solving the undetermined coefficient. However, a more interesting question of whether deep learning can discover fire-new physics is still unknown. Because deep learning is inherently lack of physical interpretability, it seems counter- intuitive to adopt physics-scare model to discover new physics. We are anticipating more exciting works to provide clearer evidence in this active topic.

## Metamaterials intelligence (metamaterials for AI)

During the time when deep learning greatly expedites metamaterial design and facilitate self- directed meta-devices, metamaterials also inversely nurture AI by providing wave-based platforms. There are many terms to describe it, such as computational metamaterials, neuromorphic photonics, and neuro-metamaterials^196–199^. To unify the whole community, we denote metamaterials for AI as metamaterials intelligence, in which the computing and reasoning ability is inherently embedded into the metamaterials themselves. Suppose, for instance, a stone-like ‘metamaterials wall’ can automatically perceive the surrounding dynamics, even though it is completely passive. When EM wave impinges on the ‘metamaterial wall’, the pre-defined ability can be implemented at the speed of light^199^. In essence, metamaterial intelligence aims to control the wave behaviours of diffraction, scattering, and interference to express the allocated tasks by using the rich degrees of freedom of metamaterial structures. As opposed to conventional electronic counterparts, metamaterials intelligence has no need for the detection of EM waves and electrical signal conversion because the computing process is all executed in physical space. Whereas electronic computing currently dominates the computing community, we cannot underestimate the unique advantages brought by metamaterials intelligence in the near future, mainly manifested by the ultra-fast speed, paralleling process, and low power consumption.

As early as 1980s, researchers have managed to exploit optical analogous computing to perform signal-processing tasks using a bulky system of lenses and filter^200,201^. A simplest example is using a convex lens to perform Fourier transformation on an arbitrary image placed in its focal plane. On this basis, we can control the spectral features of the images at Fourier space, such as suppressing high-order Fourier components with a pinhole mask, and then use a second lens to perform inverse Fourier transformation on the Fourier space. Such analogue signal processors based on Fourier optics are much faster, because the speed of light is much larger than the drift velocity of electrons. However, the bulky system broadly hinders their miniaturization, and the performed tasks are very limited. Metamaterials offer a versatile and miniaturized platform to dramatically shrink the size of the processing systems to wavelength scale and enable a myriad of functional processor for specialized tasks, such as neural network, spatial differentiator, and other exciting applications.

### Wave-based neural network

**Fig. 6:**
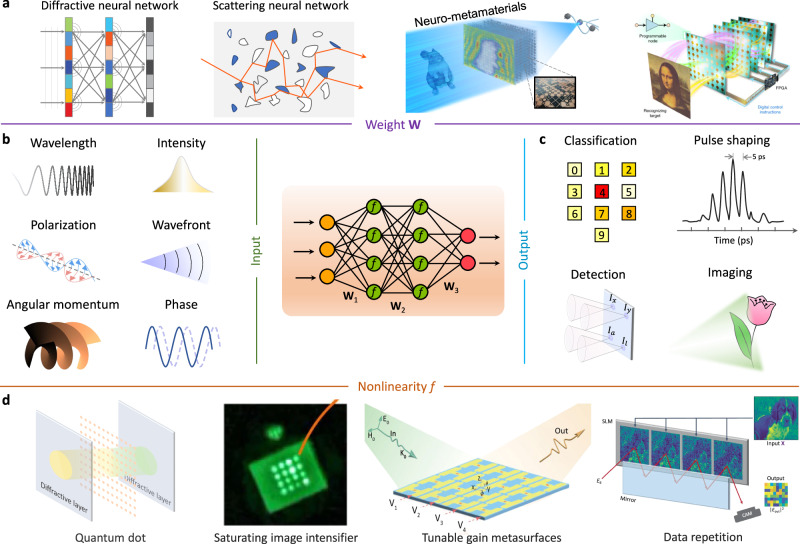
Architecture of wave-based neural network. The wave-based neural network is constructed with four crucial modules taken into account, i.e., input, output, weight, and nonlinearity. **a** The weight sculpts the input light in rigorous mathematical form, which can be physically realized by integrated photonic hardware, diffractive neural network, and scattering neural network. **b** The input is described with various optical properties, including wavelength, intensity, and polarization. **c** The output displays the results of classification, pulse shaping, detection, imaging, and other functionalities. **d** Nonlinear activation function does play a pivotal role to learn complex inference. Yet, its realization is a great challenge because optical nonlinearity is relatively weak. Currently, the related explorations include materials nonlinearity and structural nonlinearity. Panel **a** adapted with permission from ref. ^199^, CC BY 4.0; ref. ^215^, SNL; Panel **d** adapted with ref. ^220^, SNL; ref. ^221^, APS; ref. ^222^, SNL.

Wave-based neural network, an isomorphism between the physical hardware and neural network, is a fast-growing field (Fig. 6). Until now, almost all common neural networks have been (partly) mimicked with different physical principles, metamaterials structures, and network topology. Here, we provide a collection of the recent advances of wave-based neural network in different AI models with their unique strengths and pinpoint the remaining challenges for practical implementations.

#### Analogous fully-connected neural network

A fundamental building block of fully-connected neural network and modern information processing is to perform weighted summation operation or a vector-matrix multiplication operation. Metamaterials provide an inborn platform to execute wave- based matrix multiplication due to the massively parallel computing ability. There have been three main routes based on light scattering, diffraction, and interference. Metamaterials are carefully designed to bestow the transmission/reflection matrix into the light propagation behaviour in one, two, and three dimensions. First, diffractive neural network^202–210^. Diffractive neural network is composed of multiple cascaded diffractive layers with certain spatial separation. The incident wave is firstly modulated by the input layer, sequentially modulated by the diffractive (hidden) layers, and finally displays the computing results at the output layer. For each diffractive layer, the constituent element behaves like an independent neuron and interconnects to other meta-atoms of the following layers through the Rayleigh-Sommerfeld diffraction. Analogous to conventional artificial neural network, one can consider the transmission coefficient of each diffractive unit as a multiplicative “bias” term, which is a learnable network parameter that can be iteratively adjusted during the training process. The entire analogous system performs a linear matrix multiplication that transfers the input wave to the desired output wave. Researchers recently empower diffractive neural network with integration, reconfigurability, and more^173,207–210^. Ref. ^209^ reported a 3D integrated diffractive neural work for optical decryption, which is directly nanoprinted on a complementary metal-oxide-semiconductor chip by galvo-dithered two-photon nanolithography. Fan et al. proposed the concept of twisted diffractive neural work to expand the capacity limits of optical holographic storage by leveraging uncorrelated structural twist^210^. Second, scattering-based neural network^197,199^. Multiple scattering events inside inhomogeneous and bulky medium scrambles optical information as a pseudo-random matrix. The tremendous design space in the choices of materials and structural parameterizations creates a versatile platform to reshape the transmission matrix at will and perform matrix multiplication. This design necessities advanced inverse algorithms to optimize a piece of scattering media. So far, the versatility of high-contrast volumetric metamaterials has merited many applications, such as recognition, imaging, and spectral splitter. Third, interference-based neural network^211^. A typical example is connecting input and output vectors with a mesh of cascaded Mach-Zehnder interferometers (MZIs). Dating back to 1994, Reck et al. proposed a recursive algorithm that could factorize any unitary matrix into a sequence of two-dimensional matrix transformations^212^. On this theoretical basis, a programmable nanophotonic processor containing 56 MZIs in a silicon photonic integrated circuit was presented, configured to implement a matrix using singular value decomposition. Many reviews on neuromorphic photonics have been made; the interested reader is directed towards many excellent reports in the literature^52,53^.

For most of the wave-based neural network, the training is typically carried out beforehand on an electronic computer, and only the inference operation is performed optically. However, the high training burden makes such in silico training approaches fail to fully exploit the speed, efficiency, and massive parallel advantage of optical computing. For example, it takes approximately 8 h to train a five-layer diffractive ONN configured with 0.2 million neurons^202^. It takes about 140 h to design a polarization splitter with 2.4 × 2.4 μm^2^ footprint^75^. Very recently, in-situ training literatures were reported by combining reconfigurable metamaterials and photonic system with backward propagation algorithms^213–215^. In situ training can mitigate the time-consuming training process by modelling and measuring the forward and backward propagations of the optical field for its gradient calculation. Ref. ^213^ updated the diffractive modulation weights by programming a high-speed spatial light modulator. In microwave, active components (i.e., diode, amplifier) driven reconfigurable transmissive metasurfaces can be leveraged to enable real-time adjustment of diffractive weights^215^. So far, the pursuit for a more general gradient deduction algorithm and in-situ training for practical inputs is still ongoing. Another point is about the optical nonlinear activation function that plays a pivotal role to learn complex mappings and to enable a more powerful optical computing unit. Current research in optical nonlinearities explores a diverse range of phenomena and materials^216–223^, including electromagnetically induced transparency, saturation absorption, optical bistability, phase change materials, luminescent materials, and the Kerr effect (Fig. 6d). These advancements aim to enhance the functionality and efficiency of wave-based neural networks by providing robust mechanisms for nonlinear signal processing. For example, ref. ^219^ investigated a pre-sensor computing paradigm utilizing quantum dots as all-optical nonlinear activation elements. While promising, most of them typically require substantial interaction lengths and high signal powers, which limits scalability and implementation. To overcome these challenges, there is significant potential in developing reconfigurable and compact optical nonlinear devices. Such innovations could effectively emulate widely-used nonlinear activation functions like sigmoid, ReLU, and tanh, thereby advancing the capabilities of wave-based neural networks and facilitating their integration into a broader range of applications.

#### Analogous CNN

CNN is a computationally efficient model composed of convolution and pooling operation, pervasively used in computer vision, medical diagnostics, and playing board games. For the convolution portion, the mathematical essence is similar to vector-matrix multiplication. A simple way is accomplished by a modified optical 4 *f* system because the convolution between an input image and a kernel is equivalent to Fourier filtering of the input image^224^. The Fourier transform and inverse Fourier transform of an input light field containing the image information can be easily implemented by two convex lenses. For a convolution kernel, the diffractive metasurface mask is optimized and implanted in the Fourier plane of the 4 *f* setup to modify the point spread function. Ref. ^225^ reported all-optical convolutional computing by using complex-amplitude metasurfaces to arbitrarily modify the point spread function and enable functionality-unlimited kernels. Ref. ^226^ demonstrated a Fourier optics-based convolutional optical neural network integrated with multiple parallel kernels, where the convolutional kernels consist of three phases: vortex phase, random phase, and grating phase.

#### Analogous RNN

RNN is a variant of the conventional feedforward artificial neural network that is especially well-suited for sequential data like time series, speech, text, and financial data^227,228^. When it makes a decision, it considers the current input and also what it has learned from the past knowledge (internal memory). Recently, a compact inhomogeneous medium was inversely designed to learn complex features in temporal data^229^. It identifies a correspondence between the wave dynamics and RNN in theory, with an idealistic assumption of intensity-dependent wave speed. As a demonstration, vowel was theoretically classified when the waveforms propagate through it, achieving a performance that is comparable to a standard RNN. To simplify the implementation of RNN, reservoir computing has recently gained much attention, which consists of the input layer, the reservoir, and the output layer. The reservoir has randomized connections, transforming an input signal into spatiotemporal states in a higher-dimensional space. In the training phase, the input connections and reservoir internal connections remain unaltered while only the output connections are adjusted. There are two popular approaches to realize photonic reservoir computing, spatially-extended reservoirs consisting of spatially distributed nonlinear nodes^230^ and delay-based reservoirs consisting of a single nonlinear node multiplexed in time^231,232^. Recently, a new strategy based on the spatially scalable photonic library of digital micromirror devices (DMDs) and spatial light modulator (SLM) has been presented. As reported in ref. ^233^, Rafayelyan et al. showed that strong scattering media play a key role in reservoir computing to guarantee stochastic coupling weights among the numerous photonic nodes as well as parallel processing in the network. Prediction tasks in multidimensional large chaotic systems have been demonstrated in their large system with excellent accuracy and achieved a relatively high speed with low power consumption. The proposed network has demonstrated the potential scalability and capability of processing larger datasets.

#### Analogous SNN

SNN is studied to emulate biological models of neurons to carry out the computation, often referred to as the third generation of neural networks. Compared with ANN and RNN, neurons in SNN communicate with each other with discrete electrical signals called spikes and work in continuous time. Only when their membrane potential reaches the threshold, neurons are activated. Due to the functional similarity to the biological neural network, SNN can embrace the sparsity found in biology and are highly compatible with temporal code. More researchers are now engaged in training algorithms and photonics implementation of SNN^234–237^. In 2016, Prucnal’s research team demonstrated a unified spike processing platform based on the activated graphene fibre laser^234^. Subsequently, the research team proposed a distributed feedback-based laser structured neuromorphic photonic integrated circuit^235^, and discussed the feasible scheme of constructing programmable and cascadable photonic neural networks, including broadcast-and- weight network prototypes and coherent optical schemes. In 2019, Feldmann et al. proposed an all-optical spiking synaptic realization using phase-change materials^236^. When inputting the pulse, the phase-change materials unit on the waveguide is used for weighting in each neuron, and the microring resonator array is used as wavelength division multiplexing for summation. The phase- change materials have two states, crystalline and amorphous, controlled by the input optical power. For the amorphous state, the synaptic waveguide is highly transmissive and can achieve strong connections between neurons. In the crystalline state, most of the light transmitted to the phase-change materials is absorbed, leading to weak connections among neurons. As the switching of the phase-change materials cell only occurs above a certain threshold power, the neuron only generates an output pulse (spike) if the weighted sum of the input power exceeds this threshold. Thus, the system naturally emulates the basic integrate-and-fire functionality of a biological neuron.

### Wave-based mathematical operation

#### Differentiation and integration

**Fig. 7:**
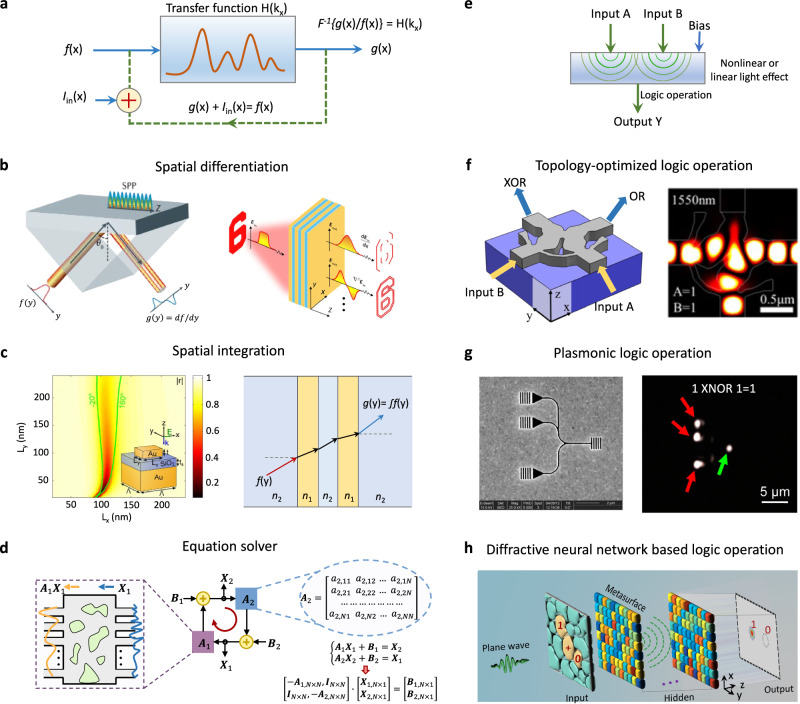
Wave-based mathematical operation and logic operation. **a** Transfer function of a linear operator in *k* space. By linking the output *g*(x) to the input *f*(x), the close-formed system can be explored to solve mathematical equation. **b** A spatial analogue differentiator based on a thin metallic film supporting SPPs and planar photonic chip. **c** Spatial integration based on a reflective metasurface array and resonant tunnelling through a dielectric slab waveguide. **d** A metamaterial platform solving integral equations of general form. **e** Logic operation based on constructive /destructive interference effects, including linear and nonlinear interference between the input light signals. **f**Topology-optimized ultracompact logic devices on silicon photonic platforms. **g** logic gates based on nanoscale plasmonic slot waveguides. **h** Diffractive neural network based universal logic operation. Panel **b** adapted from ref. ^238^, CC BY 4.0; ref. ^241^, CC BY 4.0. Panel **c** adapted with permission from ref. ^242^, ACS; ref. ^243^, OSA. Panel **d** adapted with permission from ref. ^247^, the Electromagnetics Academy and under CC BY 4.0. Panel **f** adapted with permission from ref. ^252^, ACS. Panel **g** adapted with permission from ref. ^255^, ACS. Panel **h** adapted from ref. ^251^, CC BY 4.0.

Differentiation and integration are two fundamental mathematical operations used in any field of science and engineering. When applied in image processing, spatial differentiation can enable edge detection to extract important information about the boundary of objects^238–241^, as shown in Fig. 7b. Implementing spatial differentiation in optics is highly demanded in many real-time and high-throughput scenarios, such as autonomous driving and satellite applications. Taking the function $f(x)$ as an example, the first-order differentiation $\partial f(x)/\partial x$ is equal to perform the transfer function ${{ik}}_{x}$ in Fourier space, and the second-order differentiation ${\partial }^{2}f(x)/\partial {x}^{2}$ has the transfer function of $-{k}_{x}^{2}$. There are many ways to mimic the transfer function by designing proper meta-structures, such as metasurfaces, photonic crystals, plasmonic structures, spin Hall effect, and topological photonics. Zhu et al. experimentally demonstrated first-order plasmonic differentiation based on a single layer of silver on a glass prism^238^. The thickness of the metallic layer is only about 50 nm, making the system miniaturized as compared to lens-based Fourier optical system. When the incident angle satisfies the phase matching condition, the incident wave strongly excites the surface plamonic polariton (SPP), leading to a dip of the transfer function spectrum that follows the transfer function of first-order differentiator. However, the device operates in a narrow spatial region with limited efficiency due to the condition of exciting the SPP, which in return, restricts the design to reflection type. To enable broadband edge detection, Zhou et al. proposed a Pancharatnam–Berry-phase metasurface sandwiched between two orthogonal linear polarizers^239^. The approach does not rely on complex layered structures or critical plasmonic coupling condition, but instead is based on spin-orbit interaction between light and the metasurface, showing versatile edge-detection capability with exceptional quality. Very recently, a planar photonic chip composed of a well-designed dielectric multilayer structure is proposed to simultaneously realize multiple spatial differentiations^241^. By engineering the nonlocality of this dielectric multilayer structure, the angle-dependent transmission along one direction in the momentum domain can match the requirements for one mathematical operation, and the other directions can be used to perform other operations. The photonic spatial differentiator provides a route for designing fast, power-efficient, compact and low-cost devices used in edge detection and information processing.

Implementing spatial integration is similar, for example, first-order integration has the transfer function of ${\left(i{k}_{x}\right)}^{-1}$ (Fig. 7c). Pors et al. proposed reflective plasmonic metasurfaces consisting of arrayed gold nanobricks atop a subwavelength thin dielectric spacer and optically thick gold film for analogue computing^242^. The gap-surface plasmons propagate within the dielectric spacer, demonstrating Fabry−Perot-like resonances that originate from the multiple reflections at nanobrick boundaries. Both first-order differentiation and integration were demonstrated at visible wavelengths. The excitation of gap-surface plasmons allows for the full phase and amplitude control of incident light; however, it must be used in reflection manner, which somewhat limits its potential uses for integration in optical systems that generally require transmission-type components. In 2017, a dielectric-based integrator was proposed^243^. For a simple dielectric slab, the transmission coefficient has a resonance peak at the incident angle for which the mode of the structure is excited. Around this resonance, we can approximate the transmission coefficient of the system with the Fourier-Green’s function of the spatial integration.

#### Equation solver

Mathematical equations are ubiquitous in analysing, modelling, and characterizing scientific and engineering questions. However, the slowing down and even failure of Moore’s law motivates scientists discover other disruptive approaches to solve equations. One recent work presented a fresh approach to create wave-based integral equation solver^244^, as shown in Fig. 7d. It processes an integral equation into discrete format and then designs a compact metamaterial kernel to characterize the scattering matrix using objective-first optimization. By connecting a set of output waveguides to the input waveguides of the computing kernel, the equation solution can be attained in steady state after hundreds of EM wave circulations in the closed-loop system. The difficult part of wave-based equation solver lies in how to obtain an accurate and small-footprint computing kernel. This would be especially difficult for large-scale mathematical equations. Recent works introduce the realization of arbitrary linear transformation inside meta-structures^245,246^. In ref. ^247^, Tan and co-workers decomposed high-dimensional equation into two low-dimensional matrices, mimicked by two metamaterial kernels. The capability of such tandem system has been demonstrated in solving eight complex equations. The metamaterial kernels are strongly designed with topology optimization, whose average element error is reduced to 10^−4^. The work holds great potential to extend the frontiers in integrated computing photonic circuitry, fast analogy signal processing, and optical neural network. There are also studies about other types of mathematical equations, such as differential equations and matrix inversion. For example, we express the first-order differential equations into a transfer function in spectral space, and then mimic it using Bragg photonic crystal^248^. The reliability is drastically enhanced by leveraging the unique immunity of topological insulators against structural imperfections.

### Wave-based logic operation

Optical logic operation is a fundamental building block to realize optical digital computing, meriting crucial signal processing of encoding, decoding, encryption, format conversion, switching, routing, etc. The underlying principle mainly utilizes constructive/destructive interference between the binary input light signals by virtue of linear and nonlinear effect. The implementation routes are many, including spatial light encoders, semiconductor optical amplifiers, nonlinear optical fibres, waveguides, and photonic crystals. Here we shift attention to the recent advancements; readers interested in the long-term development can refer to the recent reviews^249,250^. Ref. ^251^ introduces a simple yet universal design strategy to realize all basic optical logic operations based on diffractive neural network (Fig. 7h). The incident wave is physically encoded at the input layer, and then the compound metasurfaces (hidden layer) focus the encoded light into one of two designated areas at the output layer, which provides the logic results. On this foundation, multiple logic gates can be cascaded to enable more complex and customer-defined functionalities. However, such optical logic operation is volumetric, considerably hindering practical miniaturization requirement. Ref. ^252^ experimentally realizes an ultracompact logic device on silicon photonic platforms using topology optimization. By precisely engineering the scattering matrix, a single logic device can integrate multiple logic operations with the smallest footprint and ultralow loss small. Ref. ^253^ demonstrates the assembly of the basic logic gates for general purpose combinational logic circuits, including optical half-adder and code converter. Using polarization as the representation of digital bits is a promising approach to cascade different logical gates^254^. New materials and photonic structures will be an effective way to meet the qualitative and quantitative demands of a practice-oriented optical logic gate, such as ultralow transmission loss and logic-level restoration. Emerging nanotechnologies of nano-resonators, lithium niobates, nano-metallics, quantum dots, and even single molecules open a truly exciting but still largely unexplored possibility^255–257^.

## Outlook

The interaction between metamaterials and AI is a fast-growing field. In last few years, AI has led to an effective surrogate model of full-wave numerical simulations, a high-speed computing core that drives adaptive meta-devices, a human-machine partner that unearths hidden physics. Conversely, metamaterials provide a wave-based platform to execute task-specific tasks at the speed of light, such as neural network, mathematical operation, and logic operation. We close by identifying a longer-term outlook on this emerging field and discuss the key challenges.

Rapid progress in the development of intelligent metasurfaces is not without downsides. Many thorny questions come out, such as the de novo collection of a gigantic amount of labelled data, the repeated fine-tuning of network parameters, and the inefficiency of handling higher-degree-of -freedom objectives. From the view of dataset, in addition to hardware-accelerated simulators and experimental collectors, data augmentation techniques should be well placed to enlarge datasets with low cost, such as rotating the geometrical structures and applying equivalent physical models to generate copious variants. Also, we may advocate a more open culture of sharing and jointly contribute to a ‘global metamaterial gene bank’ incorporating canonical metamaterials samples, where researchers can freely ‘store and withdraw data’ for various demands^258^. From the view of algorithms, unsupervised and semi-supervised learning are an option because they require a relatively small number of labels and is useful for data clustering and dimensionality reduction. Even more speculatively, we shall deeply excavate the physical connection and network correlation among various tasks to integrate a heterogeneous corpus of knowledge. This is different from the stereotype of conventional transfer learning because transfer learning is more like a workhorse tool without concerning the root-cause analysis, making the performance very instable, either better or worse. Instead, we prefer to parse the physical commonalities and interpretable models to weave learning outcomes together into a tapestry of knowledge^259^. In addition, other interesting but unresolved questions are many. For example, the data-driven feature renders machine learning models vulnerable to noisy, incomplete, and biased nature of the data^145^. Even the best trained network cannot yield one hundred percent accuracy, sit also outputs unexpected anomalies; in this case, we do not have remedial strategies to correct it. For those open questions, we are still on the way to harmonize interdisciplinary researchers to initiate “off-piste” network architecture.

The forays into automating the design of metamaterials herald the dawn of intelligent meta-devices. Such paradigm goes far beyond conventional tunable metasurfaces that behave like a chicken with its head cut off, lacking the reaction mechanism to kaleidoscopic environment and external stimulus. We envisage that the final stage of meta-devices should be directed to cluttered, dynamic, and unforeseen scenes. To this end, stand-along meta-devices are not enough, which shall be equipped with a series of accessory functionalities to construct an intact perception-decision-reaction system. For the core of decision module, although the current arsenal of inverse design algorithms provides well-documented examples, they are not sufficient for intelligent meta-devices. The practical emphasis should be put on on-site learning and knowledge sharing among various scenes. Such consideration arises from the fact that surrounding environment switches quickly so that the preparation for data collection and network training is inadequate. On the other hand, in some extreme environments, the training data is not readily accessed, motivating us to supersede it with other correlated data and leverage prior domain knowledge. Moving forward, another promising frontier is the swarm of intelligent metasurfaces^260^. It dictates that distributed metasurfaces team up together to enable the integration of EM space, in lieu of stand-along metasurfaces. We posit that the number of intelligent metasurfaces and their spatial deployment shall not be fixed. Achieving this goal is difficult for orthodox neural networks because they mostly work for pre-defined and reconfiguration-bound metasurfaces. Each minute change of the swarm of metasurfaces brings the recollection of dataset and learn-from-scratch of network. Very recently, a synthetic neural network was proposed to open up an inheritance-to-assembly mixture scheme, leveraging the assemblability to broaden the adaptability of intelligent meta-devices^113–115^.

From a hardware perspective, the ability of controlling EM waves in multi-dimensions physically determines the upper limit of both intelligent metamaterials and metamaterials intelligence. Novel photonic structures, materials, active incorporations, and multiplexing methods are thus poised to be a driving force^261,262^. Often, most of metamaterials are documented to linear functionalities. With the increasing demand on wave-based computing, investigating the possibility of performing nonlinear analogue processing is a clear opportunity for the next stage of analogue computing. Furthermore, simultaneously engineering multiple properties of EM waves with metasurfaces is highly sought after, meriting numerous applications in parallel wave-based computing, high-volume wireless communication, ultrasensitive sensing, and more^263–265^. A quintessential example is that the metasurfaces composed of cross-shaped metallic patches allows for the independent manipulation of orthogonal linearly-polarized waves^265^. Gain metasurfaces are an active area to fill the shortfall of passive metasurfaces whose material loss is small, but ineluctable. They provide another degree to enhance the light-matter interaction and a versatile platform for new conceptual verification, such as optical illusion and non-Hermitian physics^266^. However, the literature about the practical realization, even the theoretical scheme, is very few. In the microwave, embedding active components into metasurfaces, such as amplifiers and tunnel diodes, is promising^220,267^.

Researchers are now attempting to bring metamaterials intelligence into real-world applications involving high-throughput and low-latency data processing. Despite a resurgence of recent interest and one decade of focused research efforts, general-purpose optical computing has yet to mature into a practical technology and most research works focus on conventional tasks with small datasets. A complete metamaterials intelligence system involves the comprehensive considerations of many factors, such as nonlinearity, cascadability, reconfigurability, integration, and more. For nonlinear activation function, besides materials nonlinearity as discussed before, it is found to generate equivalent nonlinearity by encoding the input data into metamaterials, termed as encoding linearity^222,268–270^. When cascading various wave-based computing architectures, alignment calibration error and signal attenuation would become unavoidable, and worse, such error will accumulate from layer to layer. Therefore, it is necessary to devise a more accurate and self-calibration method to describe the complex wave propagation^271^. At present, most wave-based neural networks complete the training beforehand on the electronic computer, and then implement the reasoning tasks in electromagnetism and optics. It is highly demanded to find other time-saving and energy-saving training method. Very recently, ref. ^272^ employs local gradient learning to mitigate the challenges associated with physical back-propagation calculations. Ref. ^273^ introduces a fully forward mode learning with orders-of-magnitude-faster learning processes by leveraging spatial symmetry and Lorentz reciprocity. To enable free-space metamaterials intelligence system, the design of high-efficiency reconfigurable transmissive and complex-amplitude metasurfaces are very challenging^274^. One may opt to insert gain into the system, for example, negative conductivity based metasurfaces have found to offer both gain and nonlinearity in microwave. All these challenges imply an increasing number of advancements in the near future.

From another perspective, constructing metamaterials intelligence that supersede conventional computing in any metric is challenging; instead, we can find some on-demand applications in which wave-based computing modality is the optimal, or to be complementary with electronic processors. For example, astronomical radio observation and galaxy formation exploration typically necessitate vast data to be collected and processed. Metamaterials intelligence works synergistically with conventional computing for sorting and correlating the signal looking for specific chunks of radio-patterns. Another example is that driverless vehicles and autonomous drones need to make split-second decisions to avoid collisions or deal with other unexpected events. However, this remains a big challenge for many computers running complex AI systems. Besides, many computers need large amounts of onboard computing power and sensors, all of which add to weight and are a drain on scarce battery resources. Thus, to cater to the high demands on computing speed and power saving, metamaterials intelligence may play full potential in image pre-processing, because they start from the fundamental EM scattering waves, getting rid of the need for complicated electro-optical conversion in which some information may be lost. Therefore, for some applications (e.g., imaging polarimetry) and hard-to-discover object features (e.g., surface features, shape, shading, and roughness), metamaterials intelligence may find the advantages by directly and simultaneously utilizing multiple EM wave properties, including phase, amplitude, and polarization, to reveal information that are otherwise invisible^275–277^.

The journal of intelligent metamaterials and metamaterials intelligence just starts. When we are intoxicated with the intelligent beauty of metamaterials, scientific curiosity and creativity should not be overshadowed by the dominance of deep learning. Historically, pioneering metamaterials works often originate from accidental discovery, serendipitous inspiration, and unlikeliest design. This remains out of capability of state-of-the-art deep learning algorithms as they are inherently governed by the existing templates. The practice, either intentional or unintentional, of “discovery by optimization” and “panacea by deep learning” should not be advocated. In the more distant future, we may break the cage to guide deep learning not target specific metrics but strive to achieve unexpected gain. On the path of intelligent metamaterials, many interesting and even unaware problems are waiting to be solved. More fruitful interactions with fundamental science and technological innovation could unleash the full impact to the real-world applications in a more detailed but less noticeable manner than before^278^.
