# Author Correction: A guidance to intelligent metamaterials and metamaterials intelligence

**DOI:** 10.1038/s41467-025-67597-5

**Published:** 2025-12-18

**Authors:** Chao Qian, Ido Kaminer, Hongsheng Chen

**Affiliations:** 1 ZJU-UIUC Institute, Interdisciplinary Center for Quantum Information, State Key Laboratory of Extreme Photonics and Instrumentation, Zhejiang University, Hangzhou, China; 2 ZJU-Hangzhou Global Science and Technology Innovation Center, Key Lab. of Advanced Micro/Nano Electronic Devices & Smart Systems of Zhejiang, Zhejiang University, Hangzhou, China; 3 Department of Electrical and Computer Engineering, Technion-Israel Institute of Technology, Haifa, Israel

**Keywords:** Materials for optics, Optical materials and structures

Correction to: *Nature Communications* 10.1038/s41467-025-56122-3, published online 29 January 2025

In the version of this article initially published, there was a missing milestone in the Fig. 3a timeline, where “2018: Metasurface inverse design^104^” (ref. 104: Malkiel, I. et al. *Light Sci. Appl*. 10.1038/s41377-018-0060-7 (2018)) was not listed. The figure is now amended in the HTML and PDF versions of the article.

